# Vertical movement of adult rusty grain beetles, Cryptolestes ferrugineus, in stored corn and wheat at uniform moisture content

**DOI:** 10.1673/2006.6.11.1

**Published:** 2006-07-20

**Authors:** Fuji Jian, Digvir S. Jayas, Noel D. G. White

**Affiliations:** 1 Department of Biosystems Engineering, University of Manitoba, Winnipeg, MB, R3T 5V6. Canada; 2 Agriculture and Agri-Food Canada, Cereal Research Centre, 195 Dafoe Road, Winnipeg, MB, Canada. R3T 2M9

**Keywords:** stored grain, insect movement

## Abstract

Vertical movement and distribution of Cryptolestes ferrugineus (Coleoptera: Laemophloeidae) adults in stored wheat and corn were studied in small (0.1 x 0.1 x 1 m) and large (0.6 m diameter and 1.12 m high) columns. The adults were introduced at the top, middle, and bottom of the small columns with a uniform moisture content (wheat: 14.5 ± 0.1%, corn 13.5 ± 0.1%, 15.5 ± 0.1%, and 17.5 ± 0.1%) at 27.5 ± 0.5°C. When introduced at different locations, adults showed a similar distribution in stored grain bulk with a uniform temperature and moisture content of 14.5% for wheat or 15.5% for corn. Adults showed downward displacement over 24 h when corn moisture was lower than 15.5%, but they did not show downward displacement when moisture content was 17.5%. The upward or downward movement might partially be caused by a drift effect due to beetles sliding between seeds and the displacement of the adults might be the combined effect of walking and falling during their movement. The hydrophilic behavior plus the drift effect explain why the beetles had a faster downward dispersal in the 13.5% corn than in the 15.5% and 17.5% corn and a slight upward displacement in 17.5% corn because they were more active at the lower moisture contents. Adults had a similar movement and distribution in both the small and large wheat columns.

## Introduction

An understanding of the manner in which insects disperse and distribute within bulk grain is essential to effective insect control. The adults of the rusty grain beetle, Cryptolestes ferrugineus (Coleoptera: Laemophloeidae), the most common insect pest of stored grain in western Canada (Loschiavo, 1983), move inside a grain mass in response to the changes in environmental factors such as temperature and moisture. In stored bulk grain with evenly distributed temperature and moisture, the adults show an apparent geotactic response and move from the top of grain masses down towards the bottom in small laboratory columns (Jian *et al.*, 2004; Watters, 1969; Smith, 1978; Loschiavo, 1983; White and Loschiavo, 1986).

There are a few reports about the actual distribution of C. ferrugineus adults in stored grain bins. However, because of different experimental objectives and the sampling methods in these studies, it is not possible to explain or draw a conclusion about the effect of geotactic behavior on the movement and dispersal of the beetles in stored grain bins. For example, in a 19.2 m high bin filled with wheat and sampled one month later in September of 1974, all of the insects were found in the upper 7.2 m (Smith and Loschiavo, 1978). The reason for this distribution was that the most heavily infested boxcar was unloaded last and consequently filled the upper quarter of the bin. The vertical distributions ofC. ferrugineus in newly harvested wheat in four bins (from 2.3 m to 5.5 m high) on three Kansas farms during the first two months of storage showed that the number of adults tended to decrease from top to bottom of the grain bulks in three bins. In the fourth bin, the number of beetles was highest in the middle of the grain bin (Hagstrum, 1989). Smith (1978) found the population of C. ferrugineus in a 3.8 m deep bin was very low and was distributed mainly close to the wall near the floor in two granaries (3.8 m high and 4.4 m in diameter). In the above-mentioned experiments, the beetles did not constantly show a positive geotactic behaviour.

Insects enter bulk grain from the top or bottom of stored grain bins. In Kansas, most of the insects enter farm bins at the top loading hole rather than at the eaves and floor after grain is loaded into the bins rather than before or during loading (Hagstrum, 1989). The adults then disperse from the top into the grain mass (Hagstrum, 1989). The downward movement of rusty grain beetles might help them find biologically suitable positions (Loschiavo, 1983). When a grain bin is emptied, some of the beetles can find refuges (grain residues in cracks in the wall and floors) to survive insecticide treatment and to overwinter (Jacobson and Pinniger, 1982). When the bin is filled again the beetles move up into the bulk showing a negative geotactic behavior.

It is difficult to determine the exact number of insects at every position inside a stored grain bin. Therefore, the movement and distribution of the beetles is usually determined in a small laboratory column. The movement and distribution of the insect adults is limited. The air space which the insect must go through during its movement is also influenced by the small scale because of the small overburden pressure in a short grain column. These limitations might influence insect behavior. Therefore, the important question arising from these small-scale experiments is whether or not the same responses of individuals and the same patterns of population movement will be found in large bulks of stored grain.

The aim of this research was to: 1) study how geotaxis influences C. ferrugineus adult movement when they were introduced at the top, middle, and bottom of vertical wheat and corn columns; 2) determine the distribution and movement of the adults in stored corn in different time periods at several uniform moisture contents at 27.5°C; and 3) check if there is a difference of insect distribution in small and large grain columns.

## Materials and methods

### Grain columns

**Figure 1.**
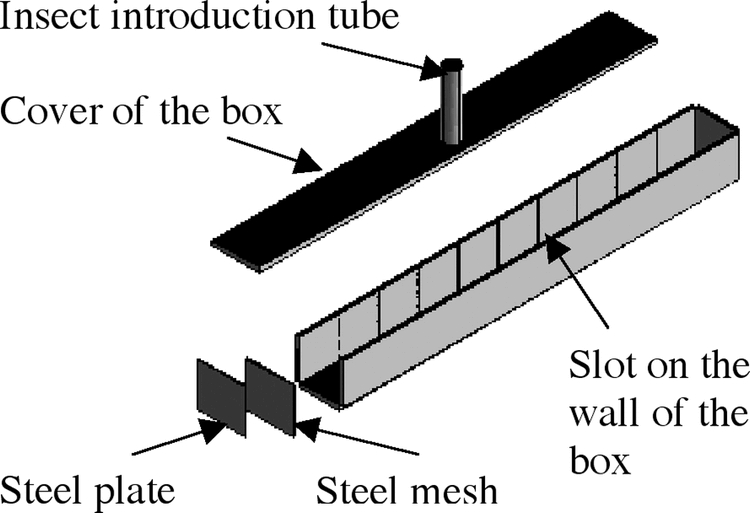
Small grain column used to test adult Cryptolestes ferrugineus movement.

Small grain columns and a large grain column were used for this study. The small grain columns (Fig. 1) were made of acrylic board (0.5 cm thickness) and had inner dimensions of 0.1 x 0.1 x 1 m. A steel mesh and steel plate (both 0.12 x 0.12 m) were fastened at the ends of the acrylic column (Fig. 1). There was a 0.2 cm diameter hole at the center of the steel plate. Insects could be introduced at one end of the grain column when the steel mesh and plate were removed. An acrylic tube (40 mm inner diameter, 4 mm thick and 160 mm long) was fixed at the middle of the acrylic cover of the grain column to permit the introduction of the insects into the center of the small grain column. During tests the tube was plugged with a wooden rod. There were nine pairs of slots on the opposite walls of the column (Fig.1). Each slot was 0.2 cm deep, 0.2 cm wide, and 10 cm long. After removing the acrylic cover, nine steel plates (101 x 110 mm) could be inserted into the slots to form 10 equal sections, each 10 cm long, 10 cm wide, and 10 cm deep.

**Figure 2.**
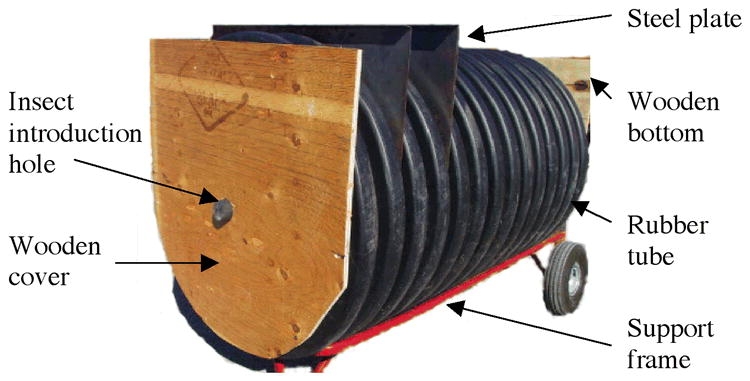
Large grain column used to test adult Cryptolestes ferrugineus movement.

The large grain column (Fig.2) was made of rubber tube with 0.6 m diameter and 1.12 m high (inner dimension). Both ends of the large grain column were made of wooden board with 2 cm thickness. There was a 5 cm hole at the center of the top cover of the large column to permit the introduction of the insects into the center of the column (Fig. 2). During insect movement the hole was plugged with a rubber stopper. There were six slots and each cut halfway through the rubber tube. During the experiment, the slots were sealed with tape. Before removing the grain out of the large column, six metal plates could be inserted into the slots to divide the tube into seven equal layers (Fig. 2).

### Grain

Corn (Pioneer 3927) and hard red spring wheat (AC Barrie) were used to fill the grain columns. Less than 0.1% waste material by mass, mainly grain husks, was found during the preliminary testing. At least 4 d before using the grain, it was moistened in a rotating drum to obtain the desired moisture content of 14.5 ± 0.1% (wet basis) for wheat, and 13.5%, 15.5% and 17.5% with standard errors of 0.1% for corn from an initial moisture content of 11 ± 1.1% and 10.5 ± 0.9% for wheat and corn, respectively. The procedure to moisten grain included: determine initial grain moisture content, calculate the amount of water to be added to reach desired moisture content, load the grain into drum, spray water on grain, rotate the drum for about 0.5 h, and place the grain into plastic bags for 4 d for moisture content distribution. Grain moisture content was determined using a standard oven-drying method by drying triplicate samples of wheat at 130ºC for 19 h and corn at 103ºC for 72 h (ASAE, 2000). Before loading the grain into columns, the grain was mixed again using the rotating drum by running the drum for 0.5 h.

### Insects

The cultures of C. ferrugineus had been reared in the laboratory for over 2 years before the experiment was started. The insects were reared at 27 ± 1ºC and 70 ± 5 % RH on whole wheat and were held in the dark during rearing and experiments. The adults (mixed sex) were less than 1 day to 2 months old at the start of each trial.

To acclimatize the adults to the testing temperature and moistures, the desired number of adults were aspirated into a vial (2.5 cm long, 0.8 cm diameter, containing about 10 g grain, loosely sealed and permeable to atmospheric gases and moisture) and the vials were kept in an environmental chamber for about 24 h before the acclimated adults were introduced into the grain column. The environmental chamber (Model: Conviron CMP3244, www.conviron.com/) was adjusted to 27.5 ± z0.5°C and the relative humidity matched the moisture content of the grain in both the small and large columns (Table 1).

**Table 1.**
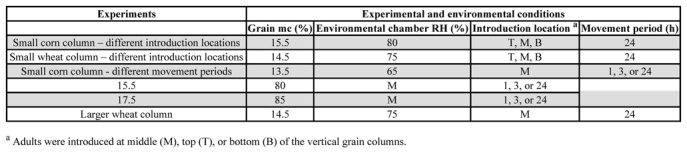
Experimental and environmental conditions of the experimental setup

### Test procedure

Four experiments were conducted in this study (Table 1) to determine insect movement and distribution in (1) a small corn column with adults introduced at top, middle, or bottom of the column, (2) a small wheat column with adults introduced at top, middle, or bottom of the column, (3) a small corn column and at different movement periods, and (4) a large wheat column. One hundred adults were introduced in a small grain column, and 850 adults were introduced in the large column.

To obtain a uniform temperature inside the grain columns, the small and large columns were kept inside the environmental chamber for at least 4 d and 14 d, respectively, before adults were introduced. After allowing insect movement for a specified period, insect movement between adjacent layers was stopped by inserting 9 steel slats into the small column or 6 steel plates into the large column (Figs. 1 and2). After grain was removed out of the columns, adults were separated from the grain by sieving. For the experiment of movement in the small column, the sieving method was the same as that used by Jian *et al.* 2003. For the experiment of movement in the large column, the wheat was passed over an inclined sieve similar to the one described by Hugstrum (1989).

Each experiment was repeated three times with new grain and different adults used for each replication. The empty grain columns were cleaned by vacuum and kept at room temperature for at least 24 h between experiments.

### Data collection and analysis

The tests of insect movement at each environmental and experimental condition were designed as a completely randomized experiment. Beetle movement and distribution under different conditions were compared by conducting the Two-Sample Location Tests and EDF statistics (SAS Institute, 2000). During this statistical analysis, the following options were selected: Wilcoxon, Median, and Kolmogorov-Smirnov (KS). The Wilcoxon option tested for difference in location, and the Median option tested for median difference in location. The Kolmogorov-Smirnov option tested differences in beetle distribution in grain columns between different environmental conditions. To control the probability of incorrectly rejecting true null hypotheses and simultaneously maintain substantial power in detecting false null hypotheses, several comparisons (refer to Table 2) of the Two-Sample-Location Test and EDF statistic were grouped together. The table-wise significance levels (Type I error) were calculated and the sequential Bonferroni test was conducted in each group.

**Table 2.**
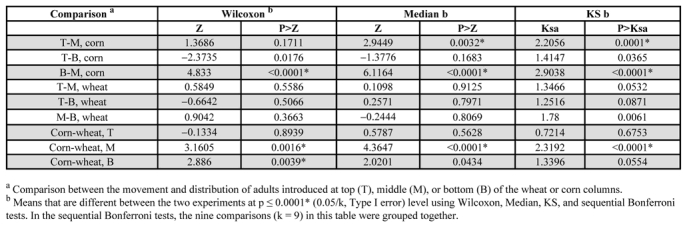
Statistical result of the movement (Wilcoxon and Median options) and distribution (Kolmogorov-Smirnov (KS) option) of Cryptolestes ferrugineus adults in wheat and corn columns at 27.5°C

During data analysis, the grain columns were divided into layers. For the small column, the top layers were sections 1 to 4 at the top end of the vertical column, and the middle layers were sections 5 and 6, and the bottom layers were sections 7 to 10. For the large column, the top layers were sections 1 to 3, the middle layer was section 4, and the bottom layers were sections 5 to 7.

To evaluate the downward movement of the adults introduced in the middle of the vertical columns, the following equation was developed:


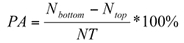


Where;

PA = the net percentage of adults moving downward or upward
N_bottom_ = total number of recovered insects in the bottom layers of a grain column
N_top_ = total number of recovered insects in the top layers of a grain column
NT = total number of recovered insects in the column

The PA value was used to test the power of downward dispersal. PA = 0 means equal number of adults moved in downward and upward directions. A higher PA value indicated more adults moved down. Adults moved up when PA<0.

## Results and Discussion

### Movement when introduced at different locations

**Figure 3.**
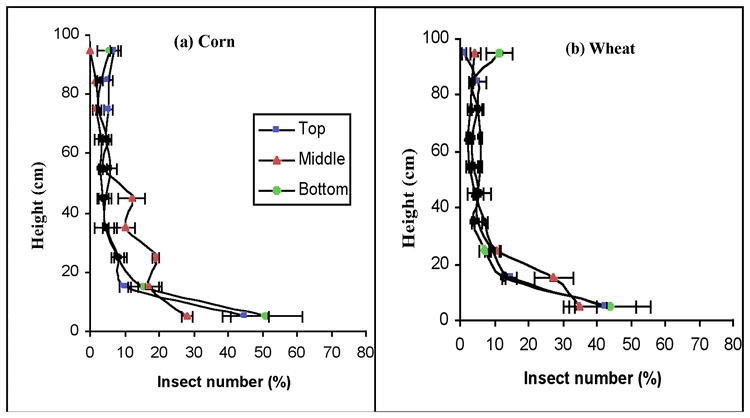
Vertical distribution of the Cryptolestes ferrugineus adults in columns of corn or wheat at 27.5±0.2°C (n = 3). The moisture content of the wheat and corn was 14.5±0.1% and 15.5±0.1% respectively. 100 adults were initially introduced at the top, middle, or bottom of the columns and movement was measured after 24 hours.

About 30% of the beetles were recovered at the bottom layer of the corn and wheat columns regardless of the introduction positions (Fig. 3a, b); most were not at the bottom. When beetles were introduced in the corn column, the distribution of those introduced at the bottom or the top were similar after 24 hours, while there was a significant difference in those introduced at the middle and top or bottom of the columns (Fig. 3a, Table 2). The movement and distribution of the adults introduced at the top of the wheat columns were the same as these introduced at the middle or bottom (Fig. 3b, Table 2); no significant differences were seen. Thus, the distribution of the adults was similar irrespective of introduction position. These results suggested that adults did not show a positive or negative geotactic behavior in a stored grain bulk with a uniform temperature at 27.5°C and uniform moisture content at 15.5% for corn or 14.5% for wheat.

During their movement among grain kernels, it might be more difficult for them to climb up than slide or fall down between the seeds. Beetles would have to control their falling. The upward or downward movement might partially be caused by a drift effect of beetles falling between seeds. The movement speed might be influenced by: 1) the spaces between seeds, which are smaller in wheat than in corn; 2) the activity of the beetles; and 3) the intensity of beetle movement. If they were more active, they might fall down faster. If they were to move up, the upward displacement would be the combined effect of moving up and falling down. If they were to move down, the downward displacement would be the combined effect of moving down and falling down. Therefore, the displacement speed of moving up would be lower than that of moving down. This may be the case in grain columns with a temperature gradient (Jian et al. 2004) or with a moisture gradient (Loschiavo, 1983). In a column with a temperature or moisture gradient, beetles may prefer to move to a biologically more suitable position that might overcome the drift effect. For example, they tend to move to warmer places (Flinn and Hagstrum, 1998). This might explain why the beetles are found in different positions within the grain bulk at different environmental conditions (Smith and Loschiavo, 1978; Hagstrum, 1989; Smith 1978). Hagstrum (1989) observed that numbers of adult C. ferrugineus beetles decrease with depth in the grain bulk of newly harvested wheat over the first 2 months of storage. The observation of Arbogast and Throne (1997) does not suggest this pattern. This contradiction might be explained by the preference for warmer and wetter grain locations and the effect of sliding. This assumption was consistent with the diffusion phenomenon of movement of C. ferrugineus in a horizontal direction in bulk wheat (Jian et al., 2005a). In a stored grain bin, dead beetles are usually found on the bottom; the reason might be that the biologically weak individuals (usually the older ones) could not control their movement and they fall downward through intergranular spaces.

The distribution of beetles introduced in the middle of a corn column was different than those that were introduced at the top or bottom of the corn columns (Fig. 3a). These results could also be caused by the sliding effect because there might be some climbing up and some sliding down when introduced into the middle of the column, while there were none climbing up when they were introduced at the top of the column. The shape and size of the intergranular space inside the corn bulk might influence the sliding effect. This could be the reason why the distribution of the beetles introduced in the middle of corn column was different than that in the wheat column (Figs. 3a and b).

Adult beetles might release pheromone during their feeding (Lindgren *et al*., 1985). Aggregation pheromones could reduce insect movement from the release point. This reduction would happen regardless of whether they were introduced at top, middle, or bottom. Because there was no significant difference between beetle movement when introduced at top or bottom, pheromone might be a minor factor influencing their movement and distribution under the experimental conditions used here.

### Movement at different periods and moisture content

**Figure 4.**
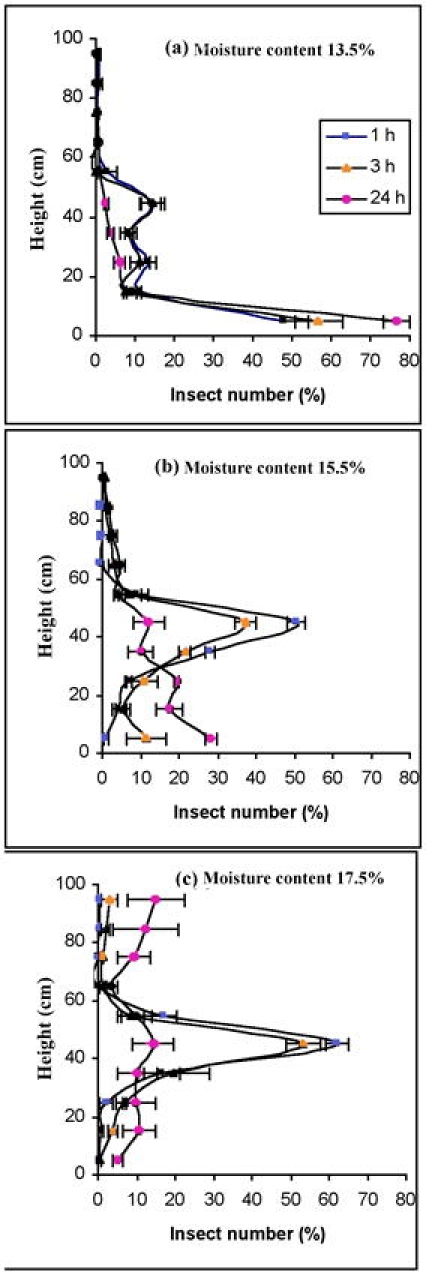
Vertical distribution of the Cryptolestes ferrugineus adults in columns of corn at 27.5 ± 0.2°C (n = 3). The moisture content of the corn was varied: a = 13.5 ± 0.2%; b = 15.5 ± 0.2%; c =17.5 ± 0.2%. 100 adults were initially introduced at the middle of the columns and movement was measured in 1, 3, or 24 h. The same data were used in Fig.4b (the 24 h data) as in Fig. 3a (data for those introduced in the middle of the column).

Beetles introduced in the middle of the small corn columns showed a downward dispersal except at a moisture content of 17.5% after a 24 h movement period (Fig. 4a, b, and c). The percent moving, the PA value, decreased with increasing moisture content (Table 3). At moisture contents of 13.5% and 15.5%, the PA value increased with longer movement time and all of the PA values were > 0, while the values decreased with longer movement times at a moisture content of 17.5% and PA values were > 0 when the movement time was at 1 or 3 h (Table 3). These results showed that beetle movement was different at different moisture content, and downward dispersal might relate to the hydrophilic behavior of the adults when the grain was dry. When grain is drier, they might more actively look for positions with biologically suitable moisture contents. In many granaries, downward dispersal would bring beetles near the floor where higher moisture often accumulates from rain or snow that enters the granary (Loschiavo, 1983). The hydrophilic behavior and greater activity in dryer grain plus the sliding downward effect explain why the beetles had a faster downward dispersal in the 13.5% corn than in the 15.5% and 17.5% moisture content corn. The 17.5% moisture content is slightly higher than the preferred moisture content of C. ferrugineus (Sinha and Watters 1985). This could explain why in the corn column with 15.5 and 17.5% moisture contents most of beetles stayed in the introduction position after 1 and 3 hours (Fig. 4b,c). By 24 hours more had moved up or down from the introduction position. Interestingly, the beetles showed a negative geotactic behavior after 24 hours when corn moisture was at 17.5% (Fig. 4c). Apparently, if beetles were allowed more time in the dryer grain column (Fig. 4a,b), the driving force to look for a wetter position, which is usually lower in a grain bin, could result in more adults moving down. This inference is consistent with the reports of Jian *et al*. (2005b).

**Table 3.**
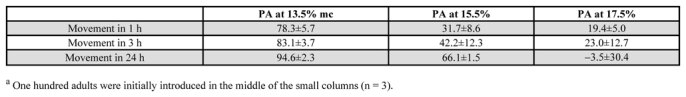
PA (net percentage of adults moving downward when PA≥0 or upward when PA≤0) values of the insect movement in the small corn column at different moisture contents ^a^

Fungi were not tested during these experiments because no obvious mould was found. However, some fungi might have multiplied in the grain columns especially at the higher moisture content. The adults might feed on the fungi (Loschiavo and Sinha, 1966) and this feeding behavior might influence their movement. Because the fungi should have been uniformly distributed in the grain column, the distribution of the adults might not have been influenced under the experimental conditions used for these experiments.

### Movement in a large column

**Figure 5.**
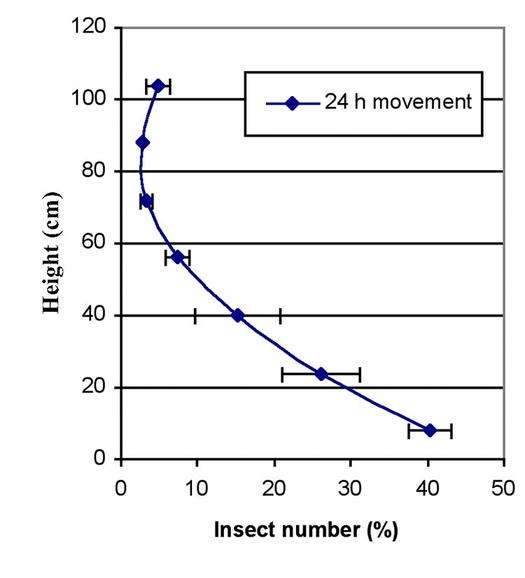
Vertical distribution of the Cryptolestes ferrugineus adults in the large wheat column at 14.5 ± 0.2% moisture content and 27.5 ± 0.2°C. 850 adults were introduced at the middle of the large column (n =3). Movement was measured after 24 h.

Adults introduced in the large wheat column had a similar distribution with those introduced in the small columns (Figs. 3b and 5). The percent moving in the small and large wheat columns was 67.4 ± 3.4% and 71.0 ± 3.1%, respectively. The T-test showed that there was no significant difference in the PA values between small and large columns (t = 0.73, p = 0.52). These results indicated that adults had a similar movement behavior in both the small and large wheat columns. Therefore, the data for insect movement and distribution in a small-scale experimental column can likely be used to predict their distribution inside a much large filled granary. This hypothesis will shortly be tested.
